# Respiratory syncytial virus glycoprotein G impedes CX_3_CR1-activation by CX_3_CL1 and monocyte function

**DOI:** 10.1038/s44298-024-00075-9

**Published:** 2024-12-05

**Authors:** Robert Meineke, Ayse Agac, Marie-Christin Knittler, Martin Ludlow, Albert D. M. E. Osterhaus, Guus F. Rimmelzwaan

**Affiliations:** Research Center for Emerging Infections and Zoonoses, University of Veterinary Medicine Hannover, Hannover, Germany

**Keywords:** Virus-host interactions, Virology, Viral immune evasion

## Abstract

The soluble form of the Respiratory Syncytial Virus (RSV) G protein (sG) bears resemblance to the chemokine fractalkine (CX₃CL1). Both RSV sG and CX_3_CL1 possess a mucin-like domain and a CX_3_C motif, exist in membrane-associated and soluble forms, and bind to the CX₃CR1 receptor expressed on immune and epithelial cells. To explore the biological significance of RSV sG and CX₃CR1 interaction, we produced wild type (WT) and CX₃C motif-deficient (CX_3_C^Mut^) RSV sG proteins and determined their effects on CX₃CR1 signaling in monocytic cells. Both CX_3_C^Mut^- and WT RSV sG failed to activate CX₃CR1 signaling directly. However, WT sG competed with CX₃CL1 for CX₃CR1 binding and reduced CX_3_CL1-induced CX₃CR1-activation, monocyte migration, and adhesion. The CX₃C motif of sG was crucial for competitive blocking of CX_3_CL1-mediated activation, as CX₃C^Mut^ sG did not affect these CX₃CR1 functions significantly. Thus, blockade of CX₃CR1 signaling by sG may allow RSV to dampen host immune responses.

## Introduction

Respiratory Syncytial Virus (RSV) is an enveloped, negative-sense, single-stranded RNA virus of the genus *Orthopneumovirus* in the *Pneumoviridae* family and is a leading cause of respiratory infections globally, particularly among infants, immunocompromised individuals and older adults^1–6^. The clinical manifestations of RSV infections vary widely, from mild, common cold-like symptoms to severe respiratory conditions such as bronchiolitis and pneumonia associated with substantial morbidity and high hospitalization rates among high-risk populations^4,5^. It is estimated that RSV causes nearly 33 million cases of acute lower respiratory infection annually in children under 5 years of age^6^. Three million of these cases require hospitalization and up to 120,000 have a fatal outcome, making RSV a leading cause of childhood mortality worldwide, especially in low and middle income countries^3,5–7^. Although RSV vaccines and human monoclonal antibodies for clinical use have become available recently^8^, these are not available for all age groups and options for antiviral treatment are limited^9–11^. The differential clinical outcome of RSV infections is still poorly understood but is most likely the result of differences in virus-host interactions^12,13^. One of the viral proteins that has been implicated to play a role in the pathogenesis of RSV infection is the G protein^7,14–16^.

The membrane-bound form of the RSV G protein is the viral attachment protein, responsible for binding to host cells via a number of molecules on the cell surface, including CX_3_CR1 through its CX_3_C motif^17^, glycosaminoglycans (GAGs) through its heparin-binding domains (HBDs)^18^, surfactant A^19^, annexin II^20^, as well as DC-Sign and L-Sign^21^ (reviewed in^7,14,22^). During infection, the G protein is also produced in a soluble form (sG)^17,23^, which is the product of a second open reading frame starting at methionine 48^24,25^. sG lacks the intracytoplasmic and the transmembrane domain, but posesses a 16 amino acid leader sequence, which facilitates its secretion from RSV-infected cells^14,24^. Several possible roles have been proposed for sG in the viral life cycle including acting as a host immune response modifier and a decoy for virus-neutralizing antibodies^26–28^. The G protein is heavily glycosylated, has a mucin-like stalk, an HBD, and a CX₃C motif within a central conserved domain^22^. N- and O-linked glycosylation is assumed to account for most of the molecular weight of the G protein and is crucial for folding and stability of the G protein^14,29^. The mucin-like stalk of the G protein contributes to its extended structure, allowing it to project from the viral surface and interact with host cell receptors more effectively^14^. The CX₃C motif is crucial for the interaction with the CX₃CR1 receptor on various cells^17^. This motif is formed by a cysteine nose via two cysteine residues at positions 182 and 186^22^, similar to the natural ligand, CX₃CL1^29^. However, the three amino acids represented by X3 in the CX₃C motif sequence differ between sG and CX₃CL1. Specifically, in CX₃CL1, the X3 sequence is Pro-Gly-Thr, whereas the corresponding sequence in RSV sG comprises of Lys-Ser-Ile. This suggests that the molecular interactions of sG and CX_3_CL1 with CX₃CR1, are different^15,17,30^.

However, the CX3C motif of the RSV G protein resembles that of CX₃CL1 structurally, and both molecules can engage CX_3_CR1. In addition, both RSV G protein and CX₃CL1 exist in two forms: a membrane-bound form that mediates attachment to cells and a soluble form that can modulate immune responses through ligand-receptor interactions^17,22^. Both RSV G protein and CX₃CL1 possess one or more mucin-like domains, which enhances their solubility and stability in the extracellular environment^23,30^. Both molecules interact with GAGs, although the specific domains responsible for this interaction may differ. The interaction of RSV G protein with GAGs facilitates viral attachment to host cells^18^, whereas in the case of CX₃CL1 it may play a role in interacting with endothelial cell surfaces^31^.

CX₃CR1, the only known receptor for CX₃CL1, is a G protein-coupled receptor (GPCR) that plays a central role in regulating host immune responses^32^. It is a unique receptor in the chemokine receptor family due to its exclusive ligand CX₃CL1, which functions as a chemokine and an adhesion molecule depending on its localization^33,34^. CX₃CR1 comprises seven transmembrane domains typical of GPCRs, with an extracellular N-terminus that binds to its ligand and an intracellular C-terminus involved in signaling upon ligand binding^33^. The interaction of CX₃CL1 with CX₃CR1 on the surface of immune cells, such as T cells and monocytes activates multiple pathways that modulate cell chemotaxis, resulting in migration of these cells towards the source of the chemokine, cell survival, proliferation, and also cell adhesion^35,36^. CX₃CR1 signaling mediates immune cell migration by activating small GTPases like Rac and Rho, which are important regulators for actin cytoskeleton rearrangement and cell motility^37^. This increased migration directs immune cells to inflammation or infection sites^33,36^. In addition, CX₃CR1 activation leads to adhesion of immune cells to the vascular endothelium, which is a prerequisite for their transmigration into tissues^33,36,38^. This process, mediated by the activation of integrins, allows immune cells to cross the endothelial barrier and access tissue sites of infection, such as the respiratory epithelium during an RSV infection^35,36,39^. The activation of integrins and their increased affinity for extracellular matrix proteins and vascular adhesion molecules enables immune cells to reach infected cells and release antiviral cytokines to control the infection^36,40^. Moreover, the activation of PI3K/Akt pathway is inhibiting apoptosis and promoting cellular proliferation, which enhances the survival and activity of immune cells at the site of infection^34,38^.

The biological relevance of CX_3_CR1 binding by virus-associated G protein has been described in pediatric patients with RSV infection as CX_3_CR1 is expressed on ciliated airway epithelial cells and co-localizes with RSV particles and CX_3_CR1+ cells showing increased susceptibility to infection^41,42^. CX_3_CR1 is expressed in epithelial cells of both the upper and lower respiratory tract, and CX_3_CR1-positive cells were more susceptible to infection. Preventing binding of RSV to CX_3_CR1 by mutating the receptor or using CX_3_CR1-specific antibodies reduced virus replication in vitro and in vivo^43^. Because impaired CX_3_CR1 signaling is associated with increased severity of RSV infection^44,45^, balancing of the CX_3_CR1 signaling seems to be crucial for the pathophysiology during RSV infection. However, the interaction between the CX_3_C-motif of the RSV G protein and CX_3_CR1 is not the only mechanism mediating the infection of airway epithelial cells and subsequent cellular signaling. As infection persists despite antibody-mediated CX_3_CR1-blockage^41^, alternative signaling pathways as L-Sign and DC-Sign^21^, or TLR2/6^46^ have to be considered when evaluating the response to RSV G stimulation.

Although the interactions between membrane-associated RSV G and CX_3_CR1 have been investigated previously, little is known about the biological relevance of the interaction between RSV sG and CX₃CR1. In the present study, we investigated whether RSV sG protein binding to CX_3_CR1 leads to the activation of CX₃CR1 signaling pathways in a manner similar to soluble CX₃CL1 or alternatively, blocks the receptor and prevents CX₃CL1-mediated signaling. Moreover, the potential interference of CX₃CL1-mediated activation of CX₃CR1 by RSV sG and its effect on immune cell activation and function, was studied.

We have studied the effect of exposure to soluble G protein on host cells in isolation, in the absence of other viral and cellular factors. By using purified recombinant proteins, we demonstrated that RSV sG can block CX_3_CR1 activation by CX_3_CL1, thereby impairing monocyte migration and adhesion to epithelial cells. Our findings show that RSV sG has previously unknown properties that may have implications for the pathogenesis of RSV, and the host immune response to RSV infections.

## Results

This study aimed to investigate the biological relevance of the interaction between RSV sG and the CX₃CR1 receptor on monocytic cells. We wished to determine if RSV sG could directly activate CX₃CR1 or potentially could block the activation by CX_3_CL1. Therefore, recombinant wild type sG protein, and a CX₃C motif-deficient mutant were produced and characterized. The CX₃C motif (aa position 182–186) was disrupted by introducing a C186R mutation to substitute the second cysteine within the motif with an arginine as described before^47,48^. Recombinant bovine serum albumin (BSA) served as a negative control. We conducted a series of experiments to compare the biological activities of the two sG variants, focusing on their ability to bind and activate the CX₃CR1 receptor, and their effect on immune cell functions mediated by CX₃CR1 signaling such as chemotactic migration of monocytes and monocyte attachment to epithelial cells.

### Protein production and purification

**Fig. 1:**
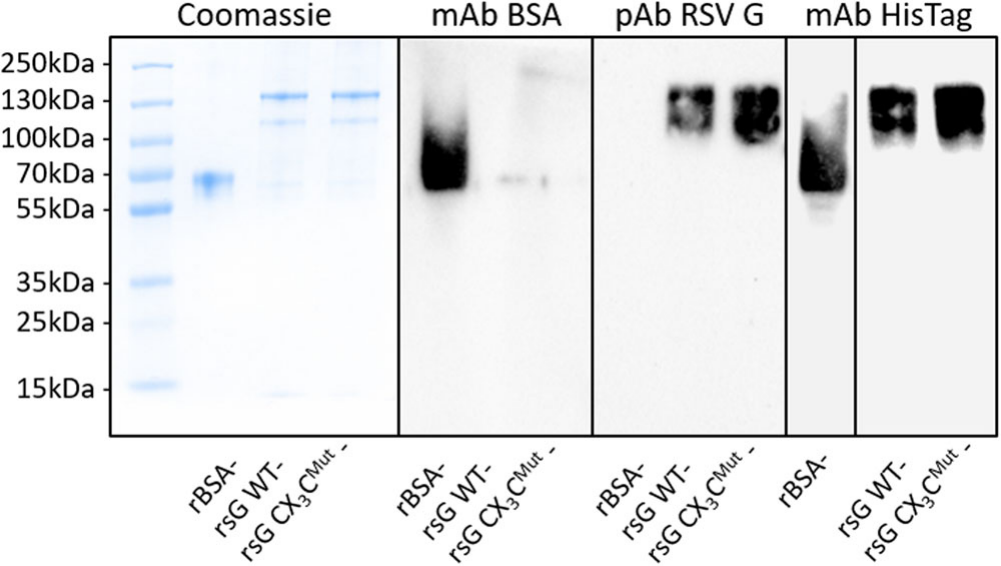
Analysis of Protein Expression and Purity of Recombinant Proteins. 1 µg purified protein samples were resolved on a 10% SDS-PAGE gel and stained with PageBlue™ coomassie solution, showing total protein content (left). Western blot analyses using rabbit anti-BSA (1:1000; A11133; Thermo Fisher) verified BSA expression, rabbit anti-RSV G glycoprotein (1:1000; 40041-T62; Sino Biological) confirmed RSV G glycoprotein expression, and mouse anti-6xHis (1:1000; His.H8; Thermo Fisher) confirmed the presence of His-tagged proteins. Secondary antibodies were applied 1:20000 (goat anti-mouse IgG, Thermo Fisher; goat anti-rabbit IgG, Thermo Fisher). All membranes were treated with SuperSignal™ Western Blot Enhancer and detected using SuperSignal™ West Pico PLUS Chemiluminescent Substrate (Thermo Fisher). Protein bands were visualized using a Chemidoc chemiluminescence imaging system (Bio-Rad). Black borders represent a division between separate blots.

His-tagged recombinant WT RSV sG, CX₃C^Mut^ sG, and BSA, were produced and purified by affinity chromatography. The purity of the proteins was assessed with SDS-PAGE and subsequent coomassie staining, and Western blot analysis (Fig. 1). Upon coomassie blue staining, distinct bands were visible that corresponded to the expected molecular weights (90–140 kDa^24,49,50^) of the purified proteins. Faint additional bands around 60 kDa were observed in RSV sG samples that made up 6.6% and 5% for WT sG and sG CX_3_C^Mut^, respectively. For Western blot analysis, both His-tag specific and antibodies specific for BSA and RSV G were used. The His-tag specific antibody detected both BSA and the RSV G proteins, whereas, only BSA or RSV sG were detected upon staining with protein-specific antibody preparations, thus confirming the identity of the respective proteins.

### RSV sG does not activate CX₃CR1 but inhibits its activation by CX_3_CL1

**Fig. 2:**
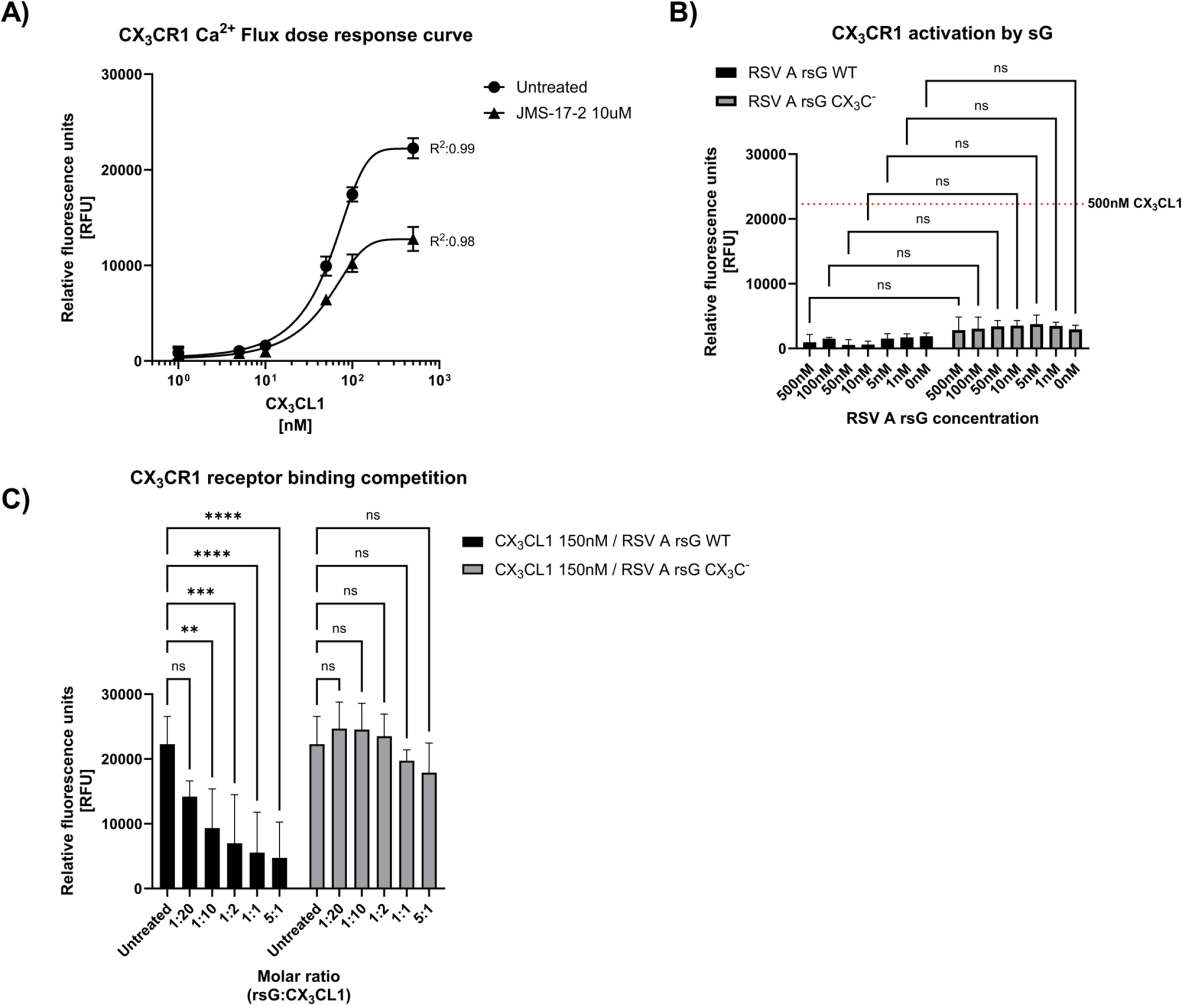
CX_3_CR1 GPCR activation assay. **A** Dose-response curve showing CX_3_CR1 activation in Chem-4 cells stimulated with increasing concentrations of CX_3_CL1 (0, 1, 5, 10, 50, 100, 500 nM). Cells pretreated with 10 µM JMS-17-2 inhibitor served as controls, with subsequent stimulation using the same concentrations of CX_3_CL1. Fluorescence intensity, indicative of calcium mobilization, was measured over 180 s. Data represent mean values ± SD (*n* = 6 from three independent experiments). **B** CX_3_CR1 activation in Chem-4 cells stimulated with recombinant RSV sG (WT or CX_3_C^Mut^) at concentrations equivalent to those in (**A**). The red dotted line indicates the mean relative fluorescence units (RFU) in response to 500 nM CX_3_CL1 (as shown in (**A**)). Data represent mean values ± SD (n = 3 from three independent experiments). *p* values were determined by Welsh t-tests compared as indicated. **C** Receptor competition assay with 150 nM CX_3_CL1 and varying molar ratios of RSV sG WT or RSV sG CX_3_C^Mut^ (5:1, 1:1, 1:2, 1:10, 1:20 CX_3_CL1) compared to untreated cells. Fluorescence intensity was measured to assess calcium flux and receptor activation. Mean values ± SD are shown (*n* = 6 from three independent experiments). *p* values were determined by Dunnett’s One-way ANOVA compared to untreated cells. ^∗^*p* < 0.05; ^∗∗^*p* < 0.01; ^∗∗∗^*p* < 0.001; ^∗∗∗∗^*p* < 0.0001.

To test whether RSV sG could activate the CX_3_CR1 receptor directly or could impair the activation of the receptor by CX₃CL1, we used a calcium flux dependent CX_3_CR1 activation assay based on Chem-4 cells overexpressing CX3CR1. Treatment of Chem-4 cells with escalating concentrations of CX₃CL1, showed a dose dependent activation of CX_3_CR1 (Fig. 2A). To confirm the specificity of this response, the Chem-4 cells were treated with JMS-17-2, a highly specific CX₃CR1 inhibitor, before exposing them to CX₃CL1. CX_3_CR1 activation reached a plateau at a CX₃CL1 concentration of ~150 nM. Pretreatment with JMS-17-2 reduced the level of receptor activation by CX_3_CL1, confirming the specificity of the detected response. The plateau remained at ~150 nM CX₃CL1, confirming that this concentration was sufficient to achieve near-maximal receptor activation (Fig. 2A). Given that Chem-4 cells overexpress CX₃CR1 and may have higher receptor levels than other cell types, this suggests that 150 nM CX₃CL1 is sufficient to achieve receptor saturation. CX_3_CR1 expression varies across different cell types and is dynamically regulated during differentiation and activation^51,52^. Therefore, we used a concentration of 150 nM CX₃CL1 in subsequent experiments to ensure consistent and robust CX₃CR1 activation across different cellular contexts.

Next, we tested if WT RSV sG and sG CX₃C^Mut^ were capable of activating CX_3_CR1. Both WT and CX₃C^Mut^ sG failed to activate CX₃CR1 signaling, and no significant difference was detected between the two proteins (Fig. 2B). With 500 nM, the highest concentration tested, relative fluorescence values were reached of 964 and 2838 RFU for RSV WT and CX₃C^Mut^ sG, respectively, which was significantly lower than for 500 nM CX₃CL1, which was used as positive control with a value of 22243 RFU (Fig. 2B).

Since RSV WT sG did not activate CX₃CR1, we next investigated whether it could compete for CX₃CR1 binding with CX₃CL1 and subsequent receptor activation. To this end, we tested 150 nM CX₃CL1 in combination with RSV sG at various molar ratios ranging from 1:20 up to 5:1 (rsG:CX₃CL1). RSV WT sG significantly reduced CX₃CR1 activation by CX_3_CL1 in a dose-dependent manner (Fig. 2C), up to a molar ratio of 1:10. In contrast, the CX₃C^Mut^ sG failed to significantly inhibit CX₃CR1 activation by CX_3_CL1 at all molar ratios tested, indicating that the inhibition achieved with WT sG is highly specific and dependent on a functional CX_3_C domain.

### RSV sG impairs migration of monocytic THP-1 cells

**Fig. 3:**
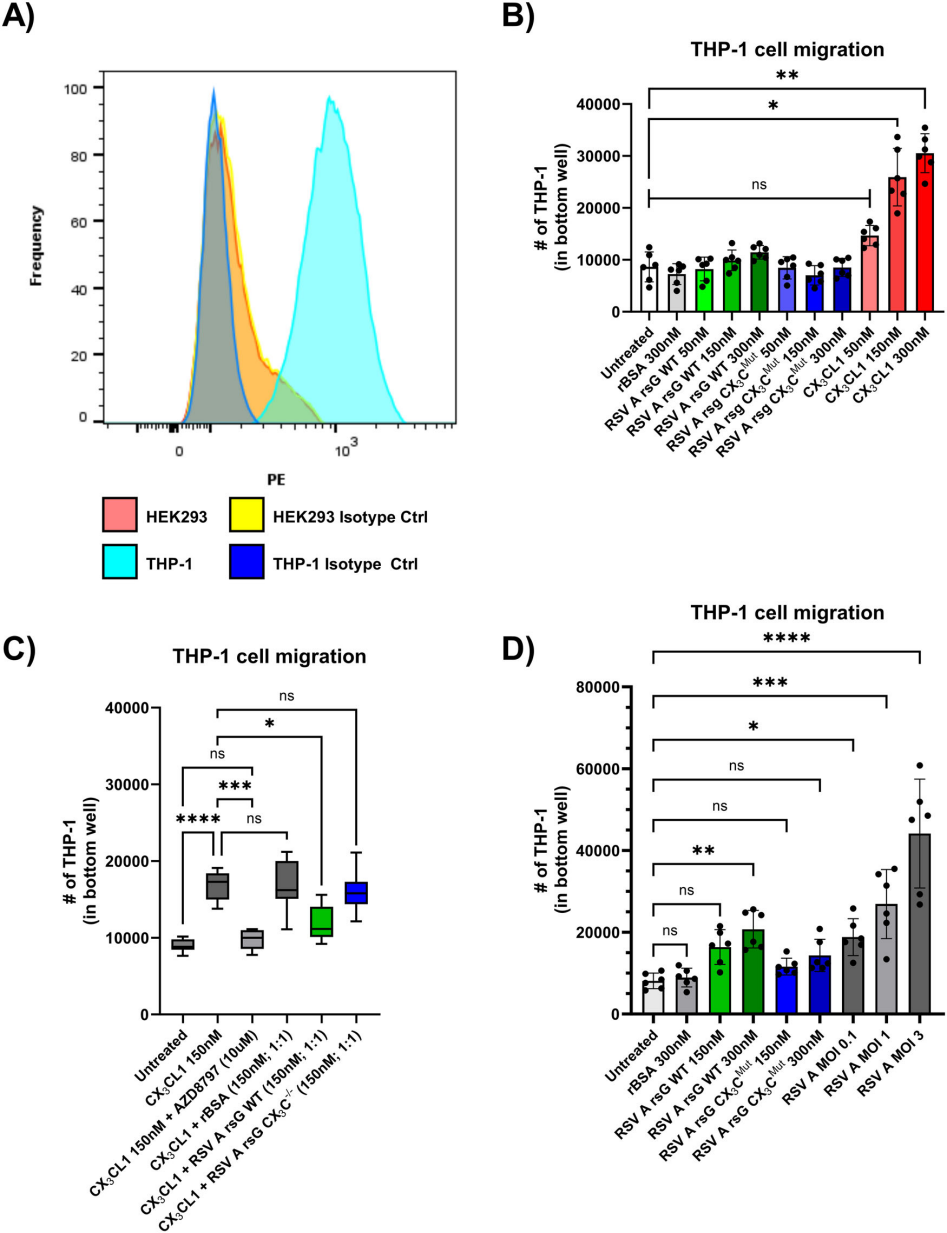
Monocyte cell migration assay. **A** Flow cytometry analysis of CX_3_CR1 expression on THP-1 cells compared to HEK293 cells (negative control). **B** Quantification of THP-1 cell migration in response to 300 nM rBSA, 50 nM, 150 nM, and 300 nM RSV A rsG WT, 50 nM, 150 nM, and 300 nM RSV A rsG CX_3_C^Mut^, 50 nM, 150 nM, and 300 nM CX_3_CL1. Data represent mean values ± SD (*n* = 6 from three independent experiments). Statistical significance was determined using One-Way ANOVA and Kruskal-Wallis tests compared to untreated cells. **C** THP-1 cell migration in response to 150 nM CX_3_CL1 in cells pretreated with 10 μM AZD8797, or equimolar ratios of rBSA, RSV A rsG WT, RSV A rsG CX_3_C^Mut^. Data represent mean values ± SD (*n* = 6 from three independent experiments). Statistical significance was determined using One-Way ANOVA and Kruskal-Wallis tests compared to 150 nM CX_3_CL1 stimulation on untreated THP-1 cells. **D** Migration of THP-1 cells induced by supernatants from A549 cells treated with 300 nM or 150 nM RSV A rsG WT, 300 nM or 150 nM RSV A rsG CX_3_C^Mut^, 150 nM rBSA, or infected with RSV A 0594 at MOI 0.1, 1, or 3. Data represent mean values ± SD (*n* = 6 from three independent experiments). Statistical significance was determined using One-Way ANOVA and Kruskal-Wallis tests compared to untreated cells. ^∗^*p* < 0.05; ^∗∗^*p* < 0.01; ^∗∗∗^*p* < 0.001; ^∗∗∗∗^*p* < 0.0001.

Monocytic THP-1 cells were used in cell migration and other assays, as a proxy for monocytes. By flow cytometric analysis we confirmed that these cells express CX_3_CR1 (Fig. 3A), indicating that they are responsive to stimuli that activate the CX_3_CR1 receptor. In this analysis, HEK293 were included as negative controls, which were confirmed to not express CX_3_CR1. To assess the biological relevance of the receptor competition by RSV sG, we performed a monocyte cell migration assay, using THP-1 cells. First, we tested whether WT RSV sG and CX₃C^Mut^ sG proteins have chemoattractant properties to attract monocytic cells. The RSV sG proteins, rBSA, or CX₃CL1 were added in equimolar concentrations (150 nM and 300 nM) to the bottom well in the cell migration assay. Migrated THP-1 cell numbers for RSV WT and CX_3_C^Mut^ sG were not significantly different to mock control or BSA, indicating that these proteins do not have chemotractant properties (Fig. 3B). In contrast, CX_3_CL1, known for its role as chemoattractant and included as positive control, significantly induced THP-1 cell migration at a concentration of 150 nM and 300 nM.

After having established that RSV sG itself does not attract monocytes, we investigated whether sG competes with CX₃CL1 for the CX₃CR1 receptor, ultimately inhibiting monocyte migration. To test this, we conducted a cell migration assay in which we tested the chemoattractant activity of CX_3_CL1 in the absence or presence of RSV sG. Again, 150 nM CX_3_CL1 exerted chemotactic effects on the THP-1 cells, which were inhibited by pretreatment with the CX₃CR1 inhibitor AZD8797, which blocks the ligand-receptor interaction, confirming that the chemotaxis is dependent on CX_3_CR1 activation. (Fig. 3C). Addition of equimolar concentrations of negative control recombinant protein BSA or CX_3_C^Mut^ sG to CX_3_CL1 (150 nM CX_3_CL1/150 nM rBSA or 150 nM CX_3_CL1/150 nM CX_3_C^Mut^ sG) in the bottom chamber did not affect the CX_3_CL1-mediated migration of THP-1. However, addition of 150 nM RSV WT sG to the bottom chamber, significantly reduced the migration, which was not observed after addition of 150 nM RSV sG with the mutated CX_3_C motif. Thus, RSV WT sG can compete with CX_3_CL1 resulting in reduced migration of monocytic cells, which is dependent on a functional CX_3_C motif (Fig. 3C).

Next, we investigated whether RSV sG can stimulate epithelial cells to secrete factors that mediate monocyte recruitment. With the culture supernatants from RSV-infected A549 cells, an MOI-dependent increase in THP-1 cell migration was observed (Fig. 3D). The culture supernatant of 300 nM WT sG-treated A549 cells was also found to induce a significant increase in cell migration. In contrast, the supernatants from sG CX₃C^Mut^ or BSA treated cells did not show a significant increase in cell migration (Fig. 3D).

### RSV sG impairs CX₃CL1/CX₃CR1-mediated monocytic cell attachment to epithelial cells

**Fig. 4:**
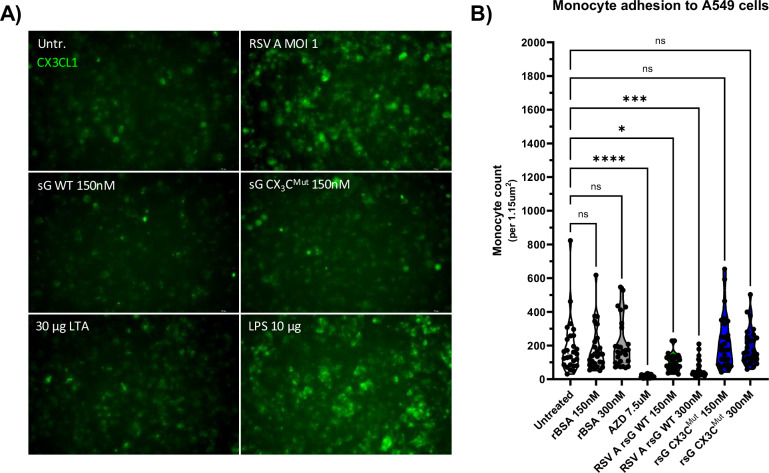
Monocyte attachment and CX_3_CL1 expression assays. **A** Immunofluorescence staining of A549 cells for CX_3_CL1 expression after 12-h treatment with 30 µg/mL LTA, 10 µg/mL LPS, 150 nM RSV A sG WT, 150 nM RSV A sG CX_3_C^Mut^, and infection with RSV A 0594 at MOI 1. CX_3_CL1-FITC antibody staining shows the level of CX_3_CL1 expression under each condition. **B** Quantification of monocyte attachment to A549 cells pretreated with 10 µg/mL LPS and subsequently exposed 7.5 µM AZD8797, 150 nM or 300 nM RSV A sG WT, 150 nM or 300 nM RSV A sG CX_3_C^Mut^, 150 nM or 300 nM rBSA. Monocyte attachment was assessed by counting calcein-positive THP-1 cells using an ImageJ automated cell counting macro adopted from ref. ^98^. Data represent mean values ± SD (*n* = 27 from three independent experiments). Statistical significance was determined using Dunnett’s One-way ANOVA compared to untreated cells. ^∗^*p* < 0.05; ^∗∗^*p* < 0.01; ^∗∗∗^*p* < 0.001; ^∗∗∗∗^*p* < 0.0001.

Given that monocytes attach via CX₃CR1 to infected cells that express CX_3_CL1 on their surface, we investigated whether RSV sG could impair monocyte attachment through binding to CX_3_CR1, similar to its effect on monocyte recruitment observed in previous experiments. First, we confirmed CX₃CL1 expression on the surface of RSV-infected A549 cells and that treatment with 150 nM of WT sG or CX₃C^Mut^ sG did not induce CX₃CL1 expression. Only infection with RSV and treatment with LPS induced CX_3_CL1 expression in A549 cells, and treatment with both variants of RSV sG, BSA or LTA did not (Fig. 4A).

In the monocyte attachment assay, CX₃CL1 expression was induced on A549 cells by LPS stimulation. THP-1 cells were labeled with the fluorescent dye calcein AM and treated with RSV WT sG, CX₃C^Mut^ sG, BSA, or the CX_3_CR1 antagonist AZD8797, and incubated with LPS-stimulated A549 cells. After 2 h, unbound cells were washed away, and after fixation of the cells, the number of fluorescently labeled THP-1 cells attached to A549 cells was assessed. As shown in Fig. 4B, both AZD8797 and WT sG treatment significantly impaired monocyte binding to CX₃CL1-expressing A549 cells. The effect was stronger for AZD8797 than for sG WT, but significant for both treatments. Specifically, the mean number of attached monocytes for the untreated control was 190 cells per field of view (1.15 µm^2^), while for AZD8797 treatment, it was significantly reduced to 17. Treatment with sG WT 150 nM and 300 nM reduced monocyte attachment to 103 and 69 cells, respectively. In contrast, treatment with sG CX₃C^Mut^ (150 nM: 232 cells/300 nM: 195 cells) and BSA (150 nM: 176 cells/300 nM: 209 cells) did not significantly impaired monocyte attachment.

## Discussion

The evolutionary pressures underlying the strict conservation of a second start codon in the RSV G open reading frame associated with the production of a soluble form of the RSV G protein is not yet completely understood. In this study, we have investigated the impact of binding of RSV sG to the chemokine receptor CX_3_CR1. We show that RSV sG cannot activate this receptor directly, but that binding to CX_3_CR1 blocks binding of, and hence activation by the natural ligand, the chemokine CX_3_CL1. This competitive interference by RSV sG impaired monocyte migration and adhesion of monocytes to epithelial cells that express CX_3_CL1 on the cell surface. The functioning of RSV sG was dependent upon the CX_3_C motif since a single amino acid substitution (C186R) in this motif abrogated all activities of sG that had been assessed^47,48^.

To be able to study the effects of RSV sG in isolation, we expressed recombinant proteins in HEK293 cells and purified them by Ni-NTA affinity chromatography. Coomassie staining of the SDS-PAGE gel revealed two proteins in the molecular weight range of 90–130 kDa with a purity of ~95%. Although the exact molecular weight of the RSV G protein is still a matter of debate, it appears to be higher for the RSV sG proteins that we purified in comparison to what has been reported previously for sG from HEp-2 cells (~90 kDa^53^,). The molecular weight of the soluble G protein has also been reported to vary between virus strains. The G protein used in the present study was derived from the RSV A strain 0594, which is of the ON1 genotype. The G gene of this virus contains a 72-nucleotide duplication, resulting in a 24 amino acid insertion in the C-terminal region of the G protein, including seven new potential O-glycosylation sites^54,55^. The G protein of the Long strain has a higher molecular weight (82–84 kDa) compared to the 18537 strain (71–78 kDa)^24,56^. These differences are primarily attributed to variations in glycosylation patterns. O-linked glycosylation, which occurs in the trans-Golgi compartment, contributes to the molecular weight differences but may have less impact than previously estimated based on electrophoretic mobility^57^. In additional, a smaller glycoprotein (43 kDa) observed in culture fluids may be a precursor or breakdown product of the G protein^58^. As cell line specific differences have been previously described^49^ and a G protein of up to 170 kDa has been derived from primary human bronchial epithelial cultures^50^, the distinct bands and their difference in molecular weight observed in the present study could also be a result of cell line dependent differences in glycosylation/maturation, oligomerisation, or truncation^50,53,59,60^. The identity of sG variant proteins and the control protein BSA, were confirmed by Western blot analysis using antibodies specific for the His-tag and RSV G or BSA, respectively.

Using the genetically engineered CX_3_CR1 reporter cell line Chem-4, we showed that RSV sG does not activate the CX₃CR1 receptor, in contrast to the chemokine CX_3_CL1, its natural ligand. The activation of CX_3_CR1 by CX₃CL1 was inhibited by CX₃CR1-specific inhibitor JMS-17-2, demonstrating that the activation by CX_3_CL1 was highly specific. Of special interest, RSV WT sG effectively competed with CX_3_CL1, resulting in impaired CX_3_CR1 activation by CX_3_CL1, up to a molar ratio of 1:10 (sG:CX_3_CL1). This indicates that the RSV sG-CX_3_CR1 interaction has high affinity. BSA and sG CX_3_C^Mut^ failed to show competition with CX_3_CL1, which indicates that the inhibition by RSV WT sG is specific and dependent on the presence of a functional CX_3_C motif.

Activation of CX_3_CR1 by CX_3_CL1 has been implicated in the functionality of monocytes, in particular their recruitment and adhesion to CX_3_CL1 expressing (RSV)-infected epithelial cells^13,31,36,38,39^. Therefore, we investigated the effect of CX_3_CL1 competition by RSV sG on migration and adhesion of monocytic cells. RSV sG not only impaired CX_3_CL1-mediated migration of THP-1 cells, it also reduced adhesion of these cells to epithelial cells that expressed CX_3_CL1. The presence of CX₃C motif in the RSV sG protein was also essential for the inhibitory effect on CX_3_CL1-mediated effector function of monocytic cells, as CX₃C-deficient sG failed to block these functions. Thus, the CX₃C motif of the G protein was essential for the assessed biological activities. In association with the viral envelope, the G protein mediates binding of RSV particles to CX₃CR1-expressing host cells^17,30,42^. In the soluble form, the CX_3_C motif of the G protein may resemble that of CX_3_CL1 structurally, but sG-binding did not activate CX₃CR1, and instead effectively blocked the receptor by preventing CX₃CL1 from binding and activating downstream signaling pathways^17^. The observation that inhibition of CX₃CR1 activation impaired immune cell functions such as migration and attachment, adds to the findings of other studies demonstrating the role of the CX_3_C motif as an attachment factor^42^ and the effect of blocking sG-CX_3_CR1 interaction in cotton rats and human ex vivo cultures^43,61^. Although several studies have shown the importance of the CX_3_C motif for RSV sG activity^15,47,62–64^, we provide the first evidence that the effects mediated by RSV sG are the result of competition with CX_3_CL1 and are not based on direct activation of CX_3_CR1.

It is of interest that RSV sG, in contrast to CX3CL1, does not activate CX₃CR1, although both molecules bind to the receptor^30,42,43,61,65^. However, the amino acid sequences of their CX₃C motifs differ. The CX3C motif of CX₃CL1 consist of the amino acids Pro-Gly-Thr^33^, while that of RSV G protein consists of Lys-Ser-Ile. These differences, may account for the differential capacity to activate the receptor^17,66^. Alternatively, sequence variation in the CX3C motif may affect folding or glycosylation of adjacent domains, which in turn, can alter the functional properties of the proteins^67^. Previous studies have shown that a specific 37-amino acid region (RSV G161-197) surounding the CX_3_C-motif responsible for CX3CR1 binding contains 24 conserved residues. Interestingly, within the CX_3_C motif, only the last of the three X amino acids, Ile185, is highly conserved, indicating that this motif alone is unlikely to represent the entire CX_3_CR1-binding site^68^.

In contrast to our findings, previous studies have shown that RSV G can induce CX_3_CR1-mediated migration in mouse leukocytes and THP-1 monocytes^17,68^. However, some differences in the experimental design may explain the contrasting results. Both studies utilized the highly lab-adapted A2 strain of RSV, while we used a contemporary RSV strain of the ON1 genotype, which better reflects currently circulating strains, although the CX_3_C-motif sequence and the 37-amino acid region (G161-197) of the A2 and 0594 strain are identical^69^. Differences at amino acid residues in the adjacent mucin-like domains may have affected the biologocal properties of the protein^66^ and may explain strain depedent differences. Tripp et al. used Vero cells for protein production, which are known to produce a G glycoprotein with altered glycosylation patterns^53^, while we used the human 293T cell line, ensuring that the glycosylation of the G protein is more consistent with that found in natural human infections^70^. Moreover, RSV sG has also been shown to interact with other receptors involved in innate immune responses such as TLR2/6^46^. By blocking binding of the G protein to the surface of immune cells^71^, the interaction with TLR2/6 and the subsequent autocrine secretion of proinflammatory chemotactically active cytokines and chemokines may be impaired. We have shown that the sG-mediated secretion of such factors induces monocytes migration and therefore could be responsible for the differences in RSV G-induced chemotaxis observed by us and others^17,68^. In addition, we have for the first time directly quantified RSV sG-mediated CX_3_CR1-signaling via quantification of calcium ion flux, while previous studies relied on chemotaxis as a proxy to quantify CX_3_CR1 activation, which may be the result of alternative pathways activated by RSV G or by RSV G-mediated autocrine stimulation.

CX₃CR1 activation typically leads to signaling via several intracellular signaling cascades, including PI3K/Akt, which promotes cell survival. Subsequently, PI3K/Akt signaling induces activation of the ERK/MAPK pathway, which regulates the activity of several transcription factors involved in cell differentiation^34^. CX₃CR1 signaling modulates calcium flux and the NF-κB pathway, contributing to inflammatory responses and cytokine production^72^. Blocking CX₃CR1 prevents PI3K/Akt activation, leading to reduced cell survival and impaired monocyte recruitment^34,73^. Similarly, inhibiting ERK/MAPK signaling affects cellular differentiation and cytokine production, while interference with NF-κB signaling impaired inflammatory responses^74^. CX₃CR1-mediated activation of the Rho family of GTPases, including Rac and Rho, is crucial for the regulation cell migration and adhesion^73,75^ and has been shown to be important antiviral host factors during RSV infection^76,77^. Rac and Rho orchestrate the dynamics of the actin cytoskeleton, central for cell movement and stable adhesion to the extracellular matrix. Rac signaling promotes the formation of lamellipodia, broad, sheet-like protrusions at the cell’s leading edge, facilitating forward movement. Rho signaling induces the assembly of stress fibers and focal adhesions, enhancing cell contraction and stabilizing attachment points^78^. The delicate balance and spatiotemporal regulation between Rac and Rho activities ensure coordinated cellular responses, enabling efficient and directed cell migration while maintaining robust attachment to the substrate. Dysregulation of these signaling pathways can lead to aberrant cell migration and attachment^79,80^. By preventing CX₃CL1 from binding to CX₃CR1, sG may block these signaling pathways, resulting in impaired monocyte migration and attachment ultimately compromising the induction of an effective inflammatory responses^81,82^.

CX₃CR1-signaling also has been shown to play a role in infections with other viruses. During chronic lymphocytic choriomeningitis virus infection, a modest decline in cytokine response was observed in CX₃CR1-deficient mice^83^. In SARS-CoV-2 infections, CX₃CR1 signaling regulated immune cell recruitment, adhesion, and subsequent cytokine responses^84,85^. Furthermore, increased expression levels of CX_3_CL1 were associated with severity of COVID-19 disease^86^. CX₃CR1 is upregulated on monocytes, dendritic cells, and macrophages during human immunodeficiency virus (HIV) infection, and subsequent activation facilitated migration and retention of these immune cells in infected tissues, influencing disease progression and viral load management^85^. The HIV envelope glycoprotein gp120 can bind to chemokine receptors (CCR5 and CXCR4), facilitating viral entry into host cells while also interfering with natural chemokine signaling^87^. Likewise, Herpes Simplex Virus (HSV) glycoprotein G has been shown to bind to chemokine receptors and inhibit their function, contributing to viral immune evasion^88^. These parallels with RSV sG suggest the existence of common evolutionary viral strategies to exploit and manipulate host chemokine systems. In the case of RSV, the inhibition of CX₃CR1 signaling by CX_3_CL1 could impair the inflammatory response, which in turn affects viral clearance^63,89^.

The impact of RSV infection and the resulting sG-CX₃CR1 interaction may vary across different age groups. RSV is particularly severe in infants and young children, often leading to bronchiolitis and pneumonia^90^, while older adults and individuals with compromised immune systems are also at high risk^4^. In infants, the immature immune system, the developing respiratory tract, and absence of previous RSV infections, contribute to the higher risk of developing severe RSV infections^91^. Of interest, age dependent differences in expression levels of CX₃CL1 and CX₃CR1 have been demonstrated, which may further contribute to differences in immune response to RSV infections in the respective age groups^36,92–94^.

Although the in vitro findings from the present study strongly support a role for sG in RSV infections, it is unclear to what extent our experimental design reflects the complex environment of infected airways in vivo. The cellular composition including endothelial, ATI, and ATII epithelial cells, fibroblasts, and tissue resident macrophages may affect the response to RSV sG stimulation via subsequent autocrine stimulation by chemotaxis inducing cytokines as CC and CXC cytokines as well as CX_3_CL1^13,95^. As shown here for monocytes, the factors secreted by A549 cells exposed to sG might also affect other cells types present in the healthy airways or recruited to the airways in response to stimulation with sG or during RSV infection. Furthermore, the levels of sG produced during a natural RSV infection are unknown, but in the in vivo microenvironment of active sites of virus replication it may be high, and sG could compete with CX₃CL1 at significant levels. Future studies should aim to quantify sG concentrations in the microenvironment of the airways during RSV infection and assess the impact of these concentrations on immune cell function in vivo or ex vivo. Additionally, our study focuses on the interactions between sG, CX₃CL1, and CX₃CR1 in the context of monocyte migration and attachment. While these are critical components of the response to infection, other factors and cell types contribute to the overall pathogenesis of RSV. For example, dendritic cells, T cells, natural killer cells, and some types of epithelial cells also express CX₃CR1 and play essential roles in antiviral immunity^33,36,52^. Future research should therefore expand to investigate the impact of sG on these cell types and its interactions with CX₃CL1 and CX₃CR1 to obtain a better understanding of all the processes affected by sG during RSV infection.

In conclusion, we show for the first time that RSV sG can engage the chemokine receptor CX_3_CR1 through its CX_3_C motif, but fails to activate the receptor. Instead, sG competes with the natural ligand and inhibits CX_3_CR1 activation by CX_3_CL1. These new insights contribute to our understanding of the pathogenesis of RSV infections and may aid development of novel intervention strategies that target the interaction between RSV sG and CX_3_CR1, which may dampen the inflammatory and antiviral responses against RSV infection.

## Material and methods

### Cell lines

Adenocarcinomic human alveolar basal epithelial cells (A549/CCL-185, ATCC) were derived from ardenocarcinomic lung tissue from a 58 year old Caucasian male. A549 cells were cultured in F-12 Medium (Ham’s F-12 Medium, Capricorn Scientific) supplemented with 10% fetal bovine serum (FBS; Gibco), 100 IU/mL penicillin (Capricorn Scientific), and 100 mg/mL streptomycin (Capricorn Scientific). HEp-2 (CCL-23, ATCC) cells were derived from a human laryngeal carcinoma of a female patient. HEp-2 cells were cultured in Eagle’s minimum essential medium (EMEM; Gibco) supplemented with 10% fetal bovine serum, 1% non-essential amino acids (NEAA; Gibco), 100 IU/mL penicillin, and 100 mg/mL streptomycin, and 1x GlutaMAX (Capricorn Scientific). The medium of A549 and HEp-2 cells was refreshed every 2–3 days, and cells were passaged upon reaching 80–90% confluency. Human monocytic THP-1 cells (TIB-202 ATCC) were derived from the peripheral blood of a 1-year-old male child with acute monocytic leukemia. THP-1 cells were maintained in RPMI-1640 medium (Capricorn Scientific) supplemented with 10% FBS, 2 mM L-glutamine (Gibco), 0.05 mM 2-mercaptoethanol (Gibco), and 100 IU/mL penicillin, and 100 mg/mL streptomycin. These cells were kept in suspension, and passaged by dilution with fresh medium when the cell density reached 1 × 10^6^ cells/mL. HEK293T (CRL-3216, ATCC) cells were cultured in DMEM (Dulbecco’s Modified Eagle Medium, Capricorn Scientific) supplemented with 10% FBS, 2 mM L-glutamine, 100 IU/mL penicillin, and 100 mg/mL streptomycin and passaged upon reaching 80–90% confluency. Chem-4 cells (Ready-to-Assay™ CX₃CR1 Chemokine Receptor Cells) were provided in Ready-to-Assay™ format (DiscoverX) and cultured according to the manufacturer’s instructions in assay-specific medium. The Chem-4 CX3CR1 cell line (RRID: CVCL_KS82) is a cell line of a undisclosed cell type derived from Rattus norvegicus (rat), engineered to express the human CX3CR1 receptor. These cells were used directly for assays without the need for passaging. All cell lines were cultured at 37 °C in a humidified atmosphere containing 5% CO₂. The identity of A549, HEp-2, THP-1, and HEK293T cells was confirmed by short tandem repeat (STR) genotyping (Microsynth).

### Plasmids and constructs

All plasmids utilized in this study were based on the pcDNA3.1(+)-C-6His backbone (Thermo Fisher Scientific) and generated by Genscript. Unmodified pcDNA3.1(+)-C-6His plasmid without insert served as a vector control. Another control plasmid was engineered to express Bovine Serum Albumin (BSA, NCBI Reference Sequence: NP_851335.1) to evaluate the system’s efficacy in protein production and use of non-relevant control protein in experiments. In addition, the gene encoding the soluble form of the RSV A G protein was inserted in pcDNA3.1(+)-C-6His based on the sequence of the RSV A strain 0594 (^69^, GenBank accession no. MW582528.1). The ON1 genotype of this strain is characterized by an 72-nucleotide duplication in the C-terminal region of the G gene^55^. For the expression of the soluble form of RSV sG, the first 144 nucleotides of the RSV G gene, including the transmembrane region, were deleted. Thus, the coding sequence of sG started from the second open reading frame at amino acid position M48, resulting in the naturally secreted form of the RSV glycoprotein G^24,25^. In addition to a wild type sG, a C186R mutation was introduced to eliminate the second cysteine within the CX₃C motif (referred to as RSV sG CX₃C^Mut^), disrupting the CX_3_C motif (aa position 182–186)^47,48^, and cloned in the pcDNA3.1(+)-C-6His backbone as described for the wild type construct above.

### Viruses

All infections in this study were performed using th RSV-A-0594 strain. This virus of the ON1 genotype carries a 24 amino acid insertion in the G protein. Virus stocks were generated by infecting HEp-2 cells at 60–80% confluency in OptiMEM (Thermo Fisher Scientific) supplemented with 100 IU/mL penicillin, and 100 mg/mL streptomycin with RSV-A-0594. Infected cells were incubated at 37 °C, 5% CO2 until cytopathic effects were visible (3–5 days). The virus was harvested by scraping the infected cells and centrifugation at 1000 × *g* for 10 min at 4 °C to remove cell debris. Supernatants were collected and mixed with 50% sterile polyethylene glycol 6000 (PEG; Carl Roth) to a final concentration of 10% PEG. Virus-PEG mix was incubated at 4 °C for 4 h on a shaker and subsequently centrifuged at 3000 × *g* for 30 min at 4 °C. The supernatant was removed, and pellets were resuspended in Halt’s balanced salt solution (Gibco) with 20% sucrose (Carl Roth). The virus was aliquoted, frozen in liquid nitrogen, and immediately stored at −150 °C until further use. Viral titers were determined by 50% tissue culture infectious dose (TCID50)/mL method on HEp-2 cells as described by Reed and Muench^96^.

### Transient transfection of HEK293T cells

HEK293T cells were transiently transfected using the calcium phosphate transfection method. Cells were seeded in 15 cm dishes at a density of 10^7^ cells/plate 24 h (h) prior to transfection to ensure 70–80% confluency at the time of transfection. For each plate, 25 µg of pcDNA3.1 plasmid DNA was mixed with 250 mM CaCl₂ in a final volume of 400 µL. This mixture was then added dropwise to an equal volume of 2X HEPES-buffered saline (HBS), pH 7.05, under vortexing to form the calcium phosphate-DNA precipitate. After a 60-min incubation at room temperature, the precipitate was added dropwise to the cells. Following an 18-h incubation, the medium was replaced with fresh DMEM containing 10% FBS to remove the calcium phosphate and cellular debris. The supernatant was collected and purified 72 h post-transfection and subjected to further purification and analysis.

### HPLC protein purification

Following transfection of HEK293T cells with RSV sG expression plasmids, the supernatants containing the secreted RSV G proteins or BSA were harvested 72 h post transfection for purification. The culture medium was first cleared of cellular debris by filtration through a 0.45 µm filter membrane. The clarified supernatant was then subjected to affinity chromatography using an Äkta Start system (Cytiva) equipped with HisTrap Excel columns (Cytiva) designed for protein purification from crude cell supernatant. The HisTrap Excel columns were equilibrated with 10 column volumes binding buffer before loading the supernatant to the column matrix. Following the binding phase, the column was washed with 50 column volumes of washing buffer containing 10 mM Imidazole to remove non-specifically bound proteins and impurities. The RSV G proteins or BSA were then eluted with an elution buffer containing 500 mM Imidazole. The eluted fractions were subjected to dialysis in 12–14 kD cutoff Pur-A-Lyzer Maxi Columns (Sigma-Aldrich) against PBS to remove Imidazole and other small molecules, thereby obtaining a concentrated, purified protein sample. All samples have been filtered via 0.45 µm syringe filter and confirmed to contain <2.5 EU/mL endotoxin by Pierce™ Rapid Gel Clot Endotoxin Assay (Thermo Fisher Scientific).

### Protein quantification and western blotting

The concentration of purified protein was determined using Quick Start™ Bradford Protein Assay (Bio-Rad) following the manufacturer’s instructions. Protein expression, purity, and identity were further assessed by Western blot analysis. Samples containing 1 µg of protein were resolved on 10% SDS-PAGE gels and stained with PageBlue™ coomassie solution (Thermo Fisher Scientific) or transferred to PVDF membranes (Cytiva). The membranes were blocked with 5% skim milk (SigmaAldrich) in TBS-T (Tris-buffered saline with 0.1% Tween20) for 1 h at room temperature to prevent non-specific binding. Primary antibodies were applied at a 1:1000 dilution: mouse anti-6xHis (Cat# MA1-21315; Thermo Fisher), rabbit anti-RSV G glycoprotein (Cat#40041-T62; Sino Biological), and rabbit anti-BSA (Cat#A11133; Thermo Fisher). After incubating overnight at 4 °C, the membranes were washed and incubated with the corresponding secondary antibodies (Cat#G-21234, goat anti-mouse IgG, Thermo Fisher; Cat#21040, goat anti-rabbit IgG, Thermo Fisher) at a 1:20000 dilution for 1 h at room temperature. The blots were developed using SuperSignal™ Western Blot Enhancer and SuperSignal™ West Pico PLUS Chemiluminescent Substrate (Thermo Fisher), and the protein bands were visualized using a Chemidoc chemiluminescence imaging system (Bio-Rad).

### CX_3_CR1 activation assay

CX₃CR1 activation was assessed using the Ready-to-Assay™ CX₃CR1 Chemokine Receptor Frozen Cells (DiscoverX). This assay is based on the measurement of calcium flux as an indicator of receptor activation. The assay procedure was carried out according to the manufacturer’s instructions. Briefly, frozen Chem-4 cells expressing the CX₃CR1 receptor were thawed and seeded into 96-well plates. The cells were incubated for 24 h at 37 °C in a humidified incubator with 5% CO₂ to allow them to adhere and recover. After 24 h, the cells were loaded with the Fluo-8® AM calcium-sensitive dye (AAT Bioquest) for 1 h at 37 °C. To assess the assay specificity, Chem-4 cells were additionally treated with 10 µM of the CX_3_CR1 inhibitor JMS-17-2 (MedChemExpress) or the corresponding concentration of DMSO for the generation of the dose response curve. After incubation, the cells were washed to remove excess dye and stimulated with recombinant CX₃CL1 (Peprotech) and/or recombinant proteins produced in HEK293T cells as described above. Calcium flux was measured directly after addition as an increase in fluorescence using a Tecan Spark microplate reader (Tecan) over a time course of 180 s at a resolution of 1 measurement per second. Peak signal was determined and analyzed to determine the relative activity by comparing the fluorescence intensities, indicative of calcium mobilization in response to CX₃CR1 activation.

### Flow cytometry to test CX₃CR1 expression

To analyze CX₃CR1 expression on THP-1 cells, we performed flow cytometry using a BD LSRFortessa™ X-20. THP-1 cells and HEK293 cells (as a negative control) were harvested and washed with PBS. Cells were blocked with Fc Block (BD Biosciences) for 10 min at 4 °C to prevent nonspecific binding. Cells were then incubated with CX₃CR1-PE antibody (Biolegend, Clone 2A9-1, Cat# 341604) diluted 1:100 in PBS with 1% BSA for 30 min at 4 °C. PE Rat IgG2b κ Isotype Control Antibody (Biolegend, Clone RTK4530, Cat# 400636) was used in parallel at the same concentration. After washing with PBS, cells were resuspended in PBS and analyzed by flow cytometry. Data were processed using FlowJo software (BD Bioscience, v10.10.0).

### Cell migration assay

Cell migration was evaluated using a Boyden chamber assay with 5 µm pore polycarbonate membrane inserts (Sarstedt). Lower chambers were filled with medium containing recombinant CX_3_CL1 (Peprotech) and/or recombinant proteins as chemoattractants. For the stimulation with conditioned A549 supernatant, A549 cells were seeded in a 24-well format and stimulated with recombinant proteins or infected with RSV-A-0594 at MOI 1 for 48 h. Supernatant was collected, filtered via a 0.45 μm membrane and used as a chemoattractant in the lower chamber in this assay. 1 × 10^5^ THP-1 cells were seeded in the upper chambers in serum-free OptiMEM medium and allowed to migrate for 6 h at 37 °C. To investigate the specificity of the migratory effect of CX_3_CL1, THP-1 cells were pretreated with 10 μM of the inhibitor AZD8797 (MedChemExppress) to block any CX_3_CR1 interaction. After 6 h, non-migrated cells were removed from the upper surface of the membrane. Migrated cells on the lower surface were detached with Accumax solution (PAN Biotech) and pooled with the content of the lower chamber. Migration was quantified by determining the cell concentration using a TC20 cell counter (Bio-Rad). The total number of cells was calculated based on the volume of medium in the bottom chamber.

### Immunofluorescence staining to detect CX_3_CL1

A549 cells were seeded at 80% confluency and treated for 12 h with RSV sG WT, RSV sG CX₃C^Mut^, rBSA, LPS, and lipoteichoic acid (LTA) (150 nM; LPS 10 μg/mL; LTA at 30 μg/mL). After 12 h, cells were fixed with 4% (w/v) paraformaldehyde (PFA) for 15 min at room temperature. The cells were then washed with PBS and blocked with 1% (w/v) BSA in PBS for 1 h. Cells were incubated with CX₃CL1-FITC antibody (Cat#CX3CL1-FITC, Thermo Fisher Scientific) diluted 1:100 in blocking buffer for 1 h at room temperature. After washing with PBS, cells were analyzed using a Leica DM-8 fluorescence microscope.

### Monocyte attachment assay

2.5 × 10^5^ THP-1 cells per well were incubated with recombinant proteins or CX₃CR1 inhibitor AZD8797 (MedChemExpress) for 1 h at 37 °C. Subsequently, cells were washed with PBS and incubated with 5 µM Calcein-AM (AAT Bioquest) in PBS for 30 min at 37 °C to fluorescently label monocytes without affecting viability or cellular processes. After the treatment, cells were washed three times with PBS supplemented with 1 mM Probenecid to block efflux of intracellular dye^97^, and transferred to a 24-well plate with confluent A549 cells with 10 µg/mL LPS pretreatment for 1 h. Co-cultures were left for 2 h at 37 °C to allow attachment of THP-1 cells to A549 cells. After 2 h, wells were washed three times with prewarmed DPBS to remove unbound THP-1 cells. The cells were fixed with 4% paraformaldehyde in PBS, and the concentration of calcein-positive THP-1 cells against the background of non-fluorescent A549 cells was assessed using a Leica DM-8 fluorescence microscope. Seven randomly selected images were taken per well and the number of fluorescent cells was determined using an ImageJ automated cell counting macro adopted from^98^.

### Quantification and statistical analysis

Statistical analyses were conducted using GraphPad Prism 10.0.3. Data are presented as means with standard deviations (SD). A *p* value of 0.05 was considered the threshold for statistical significance (ns = *p* > 0.05; ∗ = *p* ≤ 0.05; ∗∗ = *p* ≤ 0.01; ∗∗∗ = *p* ≤ 0.001; ∗∗∗∗ = *p* ≤ 0.0001). The specific tests used and the significance levels are stated in the figure legends.

## Supplementary information

Supplementary Information

## Data Availability

No datasets were generated or analyzed during the current study.
