# Supplementary material for: Respiratory syncytial virus glycoprotein G impedes CX3CR1-activation by CX3CL1 and monocyte function
Source: Npj Viruses. 2024 Dec 5;2:63. doi: 10.1038/s44298-024-00075-9 (PMC11721137; doi:10.1038/s44298-024-00075-9)
Supplement: Supplementary file 1 — Supplementary Information [file 44298_2024_75_MOESM1_ESM.docx]

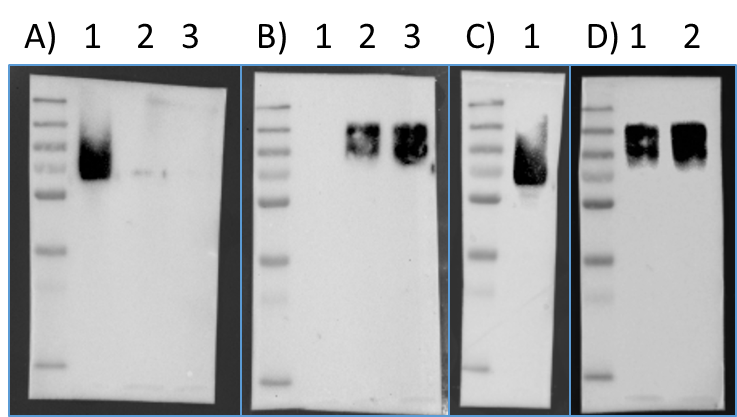


**Supplementary Figure 1. Unedited Western blot images showing protein expression and identity verification.** Full-size western blot images showing the detection of purified proteins using specific antibodies. Protein samples (1 µg) were separated on 10% SDS-PAGE gels and transferred to PVDF membranes. Images show merged colorimetric detection of the molecular weight ladder and chemiluminescent signals. (A) Western blot analysis using anti-BSA antibody showing BSA (lane 1), RSV A sG WT (lane 2), and RSV A sG CX_3_C^Mut^ (lane 3). (B) Detection using anti-RSV G glycoprotein antibody with BSA (lane 1), RSV A sG WT (lane 2), and RSV A sG CX_3_C^Mut^ (lane 3). (C) Anti-6xHis antibody detection of BSA (lane 1). (D) Anti-6xHis antibody detection of RSV A sG WT (lane 1) and RSV A sG CX_3_C^Mut^ (lane 2). Blots were developed using SuperSignal™ West Pico PLUS Chemiluminescent Substrate and visualized using a Chemidoc imaging system.
